# Effectiveness and tolerability of different therapies in preventive treatment of MOG-IgG-associated disorder: A network meta-analysis

**DOI:** 10.3389/fimmu.2022.953993

**Published:** 2022-07-26

**Authors:** Xiaofei Wang, Lingyao Kong, Zhengyang Zhao, Ziyan Shi, Hongxi Chen, Yanlin Lang, Xue Lin, Qin Du, Hongyu Zhou

**Affiliations:** ^1^ Department of Neurology, West China Hospital, Sichuan University, Chengdu, China; ^2^ Mental Health Centre and Psychiatric Laboratory, West China Hospital, Sichuan University, Chengdu, China

**Keywords:** myelin oligodendrocyte glycoprotein antibody-associated disorder (MOG-AD), treatment, relapse rate, adverse events, meta-analysis

## Abstract

**Background:**

Immunotherapy has been shown to reduce relapses in patients with myelin oligodendrocyte glycoprotein antibody-associated disorder (MOG-AD); however, the superiority of specific treatments remains unclear.

**Aim:**

To identify the efficacy and tolerability of different treatments for MOG-AD.

**Methods:**

Systematic search in Pubmed, Embase, Web of Science, and Cochrane Library databases from inception to March 1, 2021, were performed. Published articles including patients with MOG-AD and reporting the efficacy or tolerability of two or more types of treatment in preventing relapses were included. Reported outcomes including incidence of relapse, annualized relapse rate (ARR), and side effects were extracted. Network meta-analysis with a random-effect model within a Bayesian framework was conducted. Between group comparisons were estimated using Odds ratio (OR) or mean difference (MD) with 95% credible intervals (CrI).

**Results:**

Twelve studies that compared the efficacy of 10 different treatments in preventing MOG-AD relapse, including 735 patients, were analyzed. In terms of incidence of relapse, intravenous immunoglobulins (IVIG), oral corticosteroids (OC), mycophenolate mofetil (MMF), azathioprine (AZA), and rituximab (RTX) were all significantly more effective than no treatment (ORs ranged from 0.075 to 0.34). On the contrary, disease-modifying therapy (DMT) (OR=1.3, 95% CrI: 0.31 to 5.0) and tacrolimus (TAC) (OR=5.9, 95% CrI: 0.19 to 310) would increase the incidence of relapse. Compared with DMT, IVIG significantly reduced the ARR (MD=−0.85, 95% CrI: −1.7 to −0.098). AZA, MMF, OC and RTX showed a trend to decrease ARR, but those results did not reach significant differences. The combined results for relapse rate and adverse events, as well as ARR and adverse events showed that IVIG and OC were the most effective and tolerable therapies.

**Conclusions:**

Whilst DMT should be avoided, IVIG and OC may be suited as first-line therapies for patients with MOG-AD. RTX, MMF, and AZA present suitable alternatives.

## Introduction

Myelin oligodendrocyte glycoprotein (MOG) antibody-associated disorder (MOG-AD) is a demyelinating disease of the central nervous system (CNS) that causes neurological dysfunction and potential morbidity (1). The clinical symptoms of patients with MOG-AD can be present in other CNS demyelinating diseases, including acute disseminated encephalomyelitis (ADEM), optic neuritis (ON), neuromyelitis optica spectrum disorder (NMOSD), brainstem encephalitis, or multiple sclerosis (MS) (2–4). However, an increasing number of studies have shown that the clinical features, prognosis, and serum biomarkers of MOG-AD are distinct from those of NMOSD or MS (1). Therefore, MOG-AD has been recognized as a distinct disease with specific diagnostic criteria and management.

Previous studies have shown that approximately half of MOG-AD patients will experience recurrent demyelinating attacks; affected individuals may not recover from these attacks, indicating the importance of long-term prophylactic therapy in treating MOG-AD (5–7). Recently, several retrospective studies have focused on the efficacy of such therapies, such as rituximab (RTX) (6–14), mycophenolate mofetil (MMF) (7–11, 13–17), azathioprine (AZA) (6–14, 16), intravenous immunoglobulins (IVIG) (8–10, 14, 18), oral corticosteroids (OC) (8, 10, 14, 15, 18), cyclophosphamide (CTX) (9), methotrexate (MTX) (6), and disease-modifying therapy (DMT) (6, 7, 9–11, 18). However, there is no evidence regarding the optimal therapeutic strategy for preventing recurrent demyelinating attacks. Therefore, we performed this Bayesian network meta-analysis to compare and rank the efficacy and tolerability of different therapies in preventing relapse of MOG-AD.

## Materials and methods

This meta-analysis was conducted following the Preferred Reporting Items for Systematic Review and Meta-Analysis (PRISMA) guidelines.

### Search strategy

We searched PubMed, Embase, Web of Science, and Cochrane Library databases and included articles written in English until March 1, 2021. The following search keywords were used in all databases: “myelin oligodendrocyte glycoprotein,” AND “therapy,” “treatment,” “efficacy.” Detailed search strategies are listed in the (**eTable 1** in the **Supplement**). We also reviewed the reference lists of eligible studies to identify potentially relevant studies.

### Inclusion and exclusion criteria

The inclusion criteria of this study were: (1) types of studies: considering that randomized trials or prospective comparison studies on this topic do not exist, retrospective studies were included; (2) types of patients: adults or children who were diagnosed with MOG-AD in the stable phase were included, whilst patients in the acute phase were excluded; (3) types of interventions: studies using more than one type of prophylactic therapy or comparing the efficacy of drugs with non-treatment (NT) were included; (4) types of outcomes: studies reporting the incidence of relapse, annualized relapse rate (ARR), or side effects in the treatment of MOG-AD were included. Two authors (XW and ZZ) independently searched and reviewed eligible studies. Disagreements were discussed with and settled by a senior author (HZ).

### Data extraction

The following data were extracted by two authors (XW and ZZ): (1) study information, including author name, year of publication, journal name, and sample size; (2) patient information, including age, sex, and disease type; (3) treatment information, including type of therapy, dosage, and duration; and (4) reported outcomes including incidence of relapse during follow-up (primary outcome of this study), ARR, and side effects. Any disagreement between the two authors was discussed with and solved by a senior author (HZ).

### Quality assessment

The quality of eligible studies and evidence of the meta-analysis results were assessed using the Confidence in Network Meta-Analysis (CINeMA) tool (19). Specifically, in this network meta-analysis, the following six domains were assessed: (1) within-study bias, including random sequence generation, allocation concealment, blinding of participants and personnel, blinding of outcome assessment, incomplete outcome data, and selective reporting. However, these criteria have been designed for randomized controlled studies; since only retrospective studies were included in this meta-analysis, the first three criteria were classified as having an “unclear risk of bias” for all eligible studies. Eligible studies with no more than three “unclear risks of bias” were classified as “low risk”; studies with more than three “unclear risks of bias” and without “high risk of bias” were classified as “moderate risk”; studies with one “high risk of bias” were classified as “moderate risk”; studies with more than one “high risk of bias” were classified as “high risk”. (2) Reporting bias. (3) Indirectness: studies were downgraded if they only focused on the outcomes of children or adults. The study arms were downgraded if the treatments only appeared once or if the duration of treatment was less than six months. (4) Imprecision, heterogeneity, and incoherence were assessed automatically using the CINeMA software. Following these assessments, the evidence of each treatment comparison was classified as “very low”, “low”, “moderate”, or “high”.

### Data analysis

Network meta-analysis with a random-effect model within a Bayesian framework was conducted using the “gemtc” package (version 1.0-1) of R software (V.3.6.3 Foundation for Statistical Computing, Vienna, Austria). Missing mean values or standard deviations were converted using a previously published method (20). We used the Markov chain Monte Carlo (MCMC) method to obtain pooled estimates. Two Markov chains with different initial values were run separately, and those with a lower potential scale reduction factor were used. The node-splitting method was used to calculate the inconsistency of the MCMC model. The incidences of relapse and side effects were reported by odds ratios (ORs) with 95% credible intervals (CrIs). The ARR was reported by summary standardized mean differences (MD) with 95% CrIs. The rank probabilities of each treatment method were reported using the surface under the cumulative ranking curve (SUCRA). We performed meta-regression for the incidence of relapse according to the age of participants, sample size, and risk of bias of each treatment to determine whether the results were affected by these characteristics. Clustering analysis of different treatments was performed using the “cluster” package (version 2.1.2) of R and “Clusterank” of Stata (V.16, Stata-Corp, Texas, USA), and the competing treatments were clustered into meaningful groups.

## Results

### Literature search results

**Figure 1:**
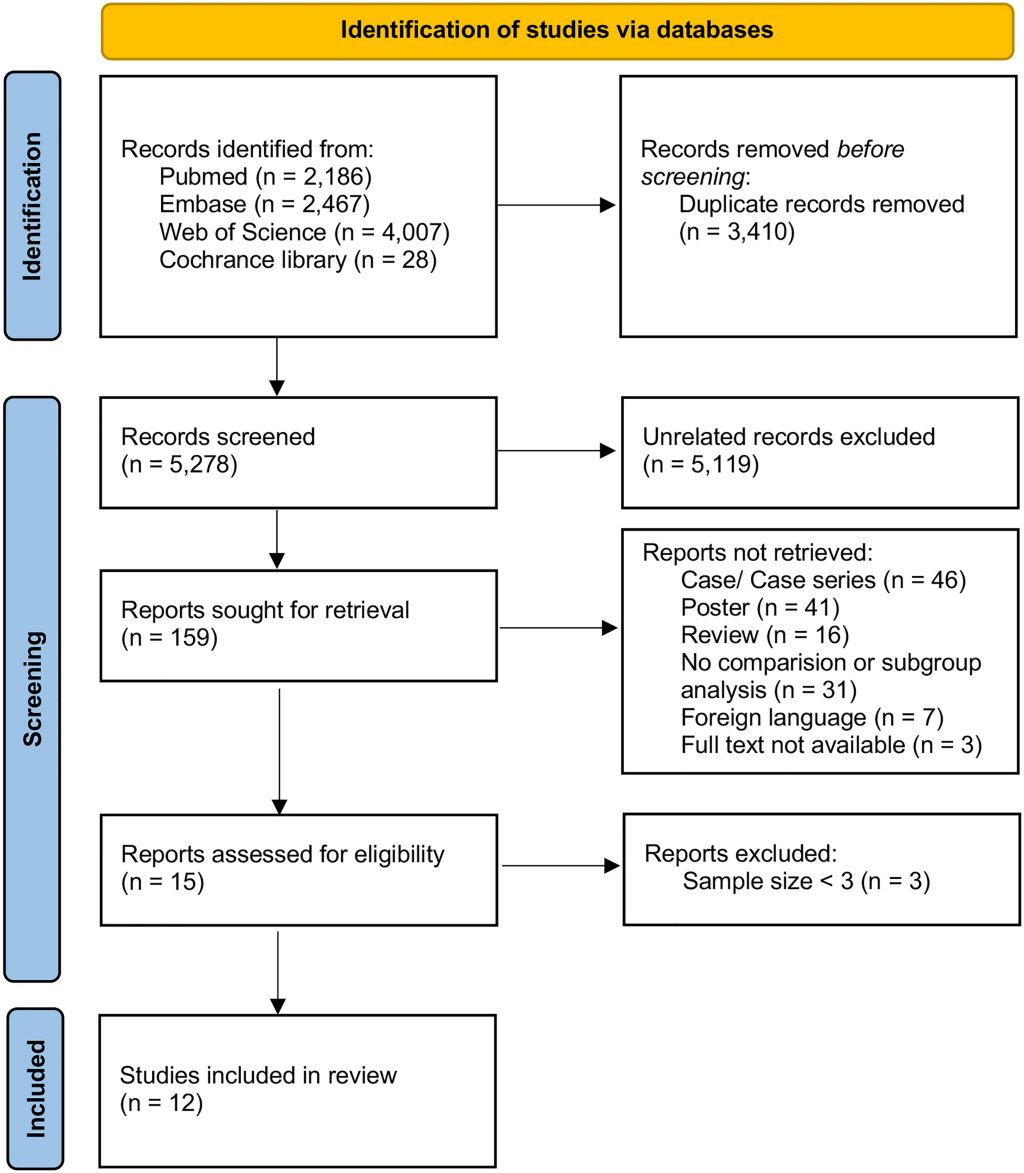
PRISMA flow diagram.

We identified 5278 citations, and 15 studies met the eligibility criteria (**Figure 1**). To enhance the reliability of statistics, treatment groups with sample sizes of less than three were further excluded. Finally, 12 studies were included in the quantitative analysis (**Table 1**), which were published between 2016 and 2021 and compared the efficacy of 10 different treatment methods in preventing relapse (6–16, 18). A total of 735 patients were included in these studies. Among them, 113 patients received AZA, 3 received CTX, 34 received IVIG, 101 received MMF, 6 received MTX, 108 received OC, 136 received RTX, 3 received tacrolimus (TAC), 59 received DMT (including interferon-β, glatiramer acetate, natalizumab, teriflunomide, fingolimod, and mitoxantrone), and 172 did not receive any treatment (NT). Four studies focused on treatment efficacy in children, two focused on adults, and six did not group by age. The minimum follow-up time was six months for most studies, except for the AZA group in the study by Pedapati et al. (16), in which it was two months. Information on the 12 enrolled studies are summarized in **Table 1**.

**Table 1:** Information of enrolled studies.

Author	Time	Journal	Age	Age at onset (year)	Gender (Male/Female)	Treatment	Sample size	Dosage	Relapse (n/%)	Adverse events	Follow-up time
Azumagawa (18)	2021	Brian & Development	Children	Mean 8.1 (SD: 3.2)	26/28	OC	39	NR	2/5.13%	NR	Mean 17 months
						IVIG	5	NR	1/20%	NR	
						DMT	5	NR	2/40%	NR	
						TAC	3	NR	2/66.67%	NR	
Li (15)	2021	Frontiers in Neurology	Children	NR	NR	OC	37	1–2 mg/kg daily for about 1 month, 0.5–1 mg/kg daily for about 2 months, and 0.25-0.5 mg/kg daily for about 3–4 months, and from 0.25 mg/kg daily to cessation for about 3–4 months. Duration of the entire treatment was more than 6 months.	9/24.32%	NR	Median 19 months (IQR: 13-27.5)
						MMF	5	NR	1/20%	NR	
Xie (8)	2021	British Journal of Ophthalmology	Both	Median 17.5 (IQR: 9.5-31.5)	54/67	AZA	6	25–50 mg/day to 2.5 mg/kg/day	2/33.33%	No AE	Median 23.8 months (IQR: 15.7-33.1)
						MMF	26	1500–3000 mg/day	5/19.23%	Liver dysfunction, infection	
						RTX	33	200 mg/time, 2 times at a 2- week interval; Retreated if CD19+ B cells reached 1.0%	10/30.3%	Infection	
						OC	4	5–10 mg/day	3/75%	No AE	
						No treatment	63	-	29/46.03%	-	Median 33.5 months (IQR: 24.2-40.4
Inan (12)	2020	Multiple Sclerosis and Related Disorders	Adults	Median 30 (range: 15-65)	7/10	AZA	9	NR	3/33.33%	NR	Median 21 months (range: 11-90)
						RTX	5	NR	2/40%	NR	
Chen (9)	2020	Neurology	Both	Median 29 (range: 3-61)	29/41	AZA	22	NR	13/59.09%	NR	Median 4.5 years (range: 1-19 years)
						MMF	19	NR	14/73.68%	NR	
						RTX	37	NR	23/62.16%	NR	
						IVIG	10	NR	2/20%	NR	
						CTX	3	NR	2/66.678%	NR	
						DMT	10	NR	10/100%	NR	
Pedapati (16)	2020	Journal of Neuroinflammation	Both	Median 59 (range: 5-57)	5/15	AZA	15	NR	12/80%	NR	Median 12 months (range: 2-108)
						MMF	3	NR	2/66.67%	NR	Median 39 months (range: 20-57)
Cobo-Calvo (7)	2019	Journal of Neuroimflammation	Adults	Median 34.1 (range: 18-67.1)	56/69	AZA	11	150 mg/day	5/45.45%	NR	Median 2.1 years (range: 0.5-12.6)
						MMF	11	2000 mg/day	3/27.27%	NR	Median 1.7 years (range: 0.5-6.8)
						RTX	26	1000 mg/every 6 months	7/26.92	NR	Median 1.7 years (range: 0.5-4.9)
						DMT	9	B-Interferon (1.a/1.b): 30 mcg/250 mcg Alternate days Glatiramer acetate: 30 mg/day Natalizumab: 300 mg/month	7/77.78%	NR	Median 3.7 years (range: 1.0-14.7)
						No treatment	59	-	28/47.46%	NR	NR
Zhou (13)	2019	Multiple Sclerosis and Related Disorders	Children	Median 5.38 (range: 2.33–12.75)	10/13	AZA	3	NR	0/0	NR	0.75-1.25 years
						MMF	3	NR	1/33.33%	NR	0.58-1.58 years
						RTX	8	NR	5/62.5%	NR	0.50-1.83 years
Hacohen (10)	2018	JAMA Neurology	Children	Median 7.0 (range: 1.5-7.9)	36/66	AZA	20	NR	10/50%	NR	Median 5.0 years (range: 3.0-9.0)
						MMF	15	NR	8/53.33%	NR	
						RTX	9	NR	6/66.67%	NR	
						IVIG	12	NR	4/33.33%	NR	
						DMT	16	NR	15/93.75%	NR	
						OC	8	NR	5/62.5%	NR	
						No treatment	50	-	50/100%	NR	Median 5.0 years (range: 3.0-8.0)
Ramanathan (14)	2018	Journal of Neurology, Neurosurgery & Psychiatry	Both	Median 12 (range: 1-74)	19/40	AZA	4	2 - 3 mg/kg/day	2/50%	Intolerable nausea	Median 45 months (range: 12-288)
						MMF	16	1000 to 2000 mg/day	9/56.25%	No AE	
						RTX	6	1000 mg/time, 2 times at a 2- week interval; Retreated if CD19+ B cells > 0.1% or at regular 6-month intervals	4/66.67%	No AE	
						IVIG	7	2 g/kg during an induction course dose; 1 g/kg/month/infusion subsequently	3/42.86%	No AE	
						OC	20	>10 mg/day for patients >40 kg in weight; >5 mg/day for patients ≤40 kg in weight	8/40%	No AE	
Hyun (11)	2017	Journal of Neurology, Neurosurgery & Psychiatry	Both	Median 30 (range: 4–50)	8/14	AZA	6	NR	1/16.67%	NR	Median 39 months (range: 27-92)
						MMF	3	NR	1/33.33%	NR	
						RTX	3	NR	2/66.67%	NR	
						DMT	5	NR	1/20%	NR	
Jarius (6)	2016	Journal of Neuroimflammation	Both	Median 31 (range: 6-70)	13/37	AZA	17	NR	14/82.35%	No AE	Mean 75 months (SD 46.5 months)
						RTX	9	NR	7/77.78%	Allergic exanthema	
						DMT	14	NR	12/85, 71%	Leukopenia (Glatiramer acetate, B-Interferon), recurrent headache (Natalizumab)	
						MTX	6	NR	5/83.33%	Severe infection	

AE, adverse events; AZA, azathioprine; CTX, cyclophosphamide; DMT, disease-modifying therapy; IVIG, intravenous immunoglobulins; MMF, mycophenolate mofetil; MTX, methotrexate; NR, not report; OC, oral corticosteroids; RTX, rituximab; TAC, tacrolimus.

### Treatment efficacy in reducing incidence of relapse

**Figure 2:**
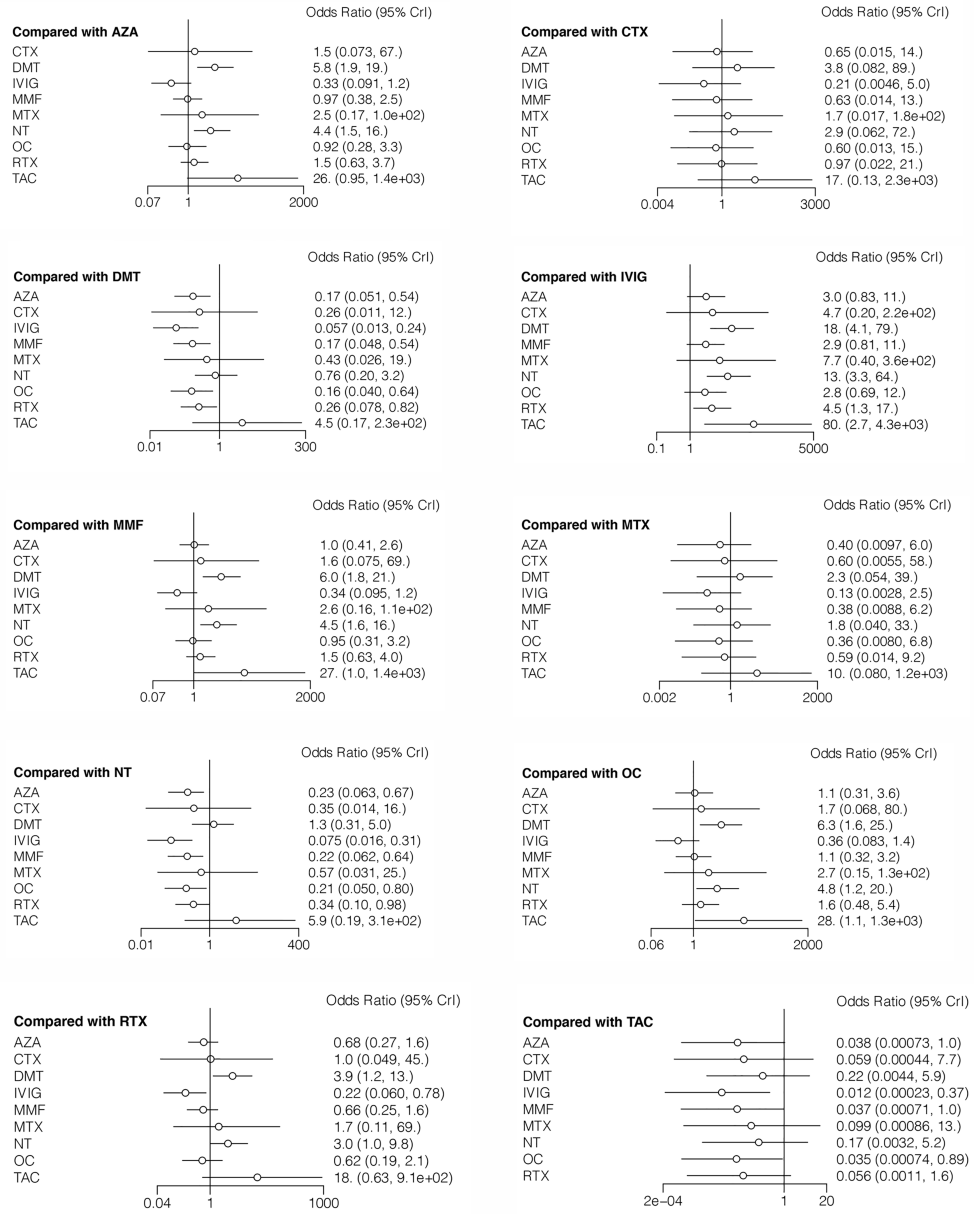
Forest plots of network meta-analysis for incidence of relapse.

The incidence of relapse during follow-up was reported in all 12 studies, with reference to 10 different treatment methods (6–16, 18). The estimated ORs with 95% CrIs of relative effectiveness are listed in **Table 2**. According to the SUCRA, IVIG was hierarchically superior to other treatments, followed by OC, MMF, AZA, CTX, RTX, MTX, NT, DMT, and TAC (**eTable 2** in the **Supplement**). A comparison between different medications is shown in **Figure 2**. IVIG, OC, MMF, AZA, and RTX were all significantly more effective than NT (ORs ranged from 0.075 to 0.34, **Figure 2**). The node-splitting model exhibited segmental inconsistency for IVIG versus NT (direct: MD=60.0, 95% CrI: 7.9 to 1.8×10 (2); indirect: MD=1.8, 95% CrI: 0.35 to 3.2; network: MD=2.6, 95% CrI: 1.2 to 4.2; *P*<.001) and IVIG versus OC (direct: MD=0.17, 95% CrI: −1.5 to 1.8; indirect: MD=4.0, 95% CrI: 1.0 to 7.5; network: MD=1.0, 95% CrI: −0.3 to 2.5; *P*=.03). However, the mixed results were consistent with the direct pairwise results. Network meta-regression was performed to adjust for the influence of age, sample size, and risk of bias, and most treatments were not significantly affected by these modifiers (**eTable 3** in the **Supplement**).

**Table 2:** Estimated odds ratio with 95% credible intervals of different treatments in reducing incidence of relapse in MOG-AD.

AZA	1.54 (0.07, 65.21)	5.81 (1.87, 19.43)	0.33 (0.091, 1.20)	0.97 (0.38, 2.46)	2.52 (0.17, 105.46)	4.36 (1.50, 15.84)	0.91 (0.28, 3.26)	1.47 (0.63, 3.74)	25.85 (0.95, 1364.46)
0.65 (0.015, 13.47)	CTX	3.80 (0.083, 87.04)	0.21 (0.0047, 4.98)	0.63 (0.015, 13.0)	1.69 (0.017, 189.83)	2.88 (0.064, 70.51)	0.60 (0.013, 14.4)	0.96 (0.023, 20.25)	16.78 (0.13, 2224.24)
0.17 (0.051, 0.54)	0.26 (0.011, 12.0)	DMT	0.056 (0.013, 0.24)	0.17 (0.048, 0.53)	0.44 (0.026, 18.968)	0.75 (0.20, 3.18)	0.16 (0.04, 0.63)	0.25 (0.078, 0.82)	4.43 (0.17, 227.45)
3.05 (0.84, 11.0)	4.70 (0.20, 213.28)	17.73 (4.13, 79.22)	IVIG	2.94 (0.81, 10.46)	7.76 (0.41, 365.47)	13.37 (3.25, 63.67)	2.78 (0.69, 12.04)	4.48 (1.27, 16.68)	78.98 (2.70, 4365.54)
1.03 (0.41, 2.65)	1.59 (0.077, 68.32)	6.01 (1.87, 20.85)	0.34 (0.096, 1.24)	MMF	2.63 (0.16, 114.85)	4.50 (1.58, 16.25)	0.94 (0.31, 3.18)	1.52 (0.63, 4.03)	26.84 (0.99, 1410.31)
0.40 (0.009, 6.00)	0.59 (0.0053, 57.72)	2.30 (0.053, 38.48)	0.13 (0.0027, 2.44)	0.38 (0.0087, 6.24)	MTX	1.74 (0.039, 32.74)	0.36 (0.0078, 6.75)	0.58 (0.014, 9.15)	9.86 (0.077, 1150.08)
0.23 (0.063, 0.67)	0.35 (0.014, 15.70)	1.33 (0.31, 5.0)	0.075 (0.016, 0.31)	0.22 (0.062, 0.64)	0.57 (0.031, 25.58)	NT	0.21 (0.05, 0.80)	0.34 (0.10, 0.98)	5.85 (0.19, 316.83)
1.09 (0.31, 3.60)	1.68 (0.069, 77.46)	6.38 (1.58, 25.0)	0.36 (0.083, 1.44)	1.06 (0.31, 3.21)	2.78 (0.15, 128.57)	4.81 (1.25, 20.0)	OC	1.61 (0.48, 5.385)	28.13 (1.11, 1355.97)
0.68 (0.27, 1.59)	1.04 (0.049, 43.69)	3.94 (1.23, 12.82)	0.22 (0.060, 0.78)	0.66 (0.25, 1.59)	1.71 (0.11, 71.48)	2.97 (1.03, 9.75)	0.62 (0.19, 2.09)	RTX	17.49 (0.63, 907.65)
0.039 (0.0007, 1.05)	0.060 (0.0004, 7.96)	0.23 (0.0044, 5.98)	0.013 (0.00023, 0.37)	0.037 (0.00071, 1.01)	0.10 (0.00087, 12.99)	0.17 (0.0032, 5.32)	0.036 (0.00074, 0.90)	0.057 (0.0011, 1.58)	TAC

AZA, azathioprine; CTX, cyclophosphamide; DMT, disease-modifying therapy; IVIG, intravenous immunoglobulins; MMF, mycophenolate mofetil; MTX, methotrexate; NT, no treatment; OC, oral corticosteroids; RTX, rituximab; TAC, tacrolimus. A deeper color indicates statistical significance.

### Treatment efficacy in reducing the ARR

**Figure 3:**
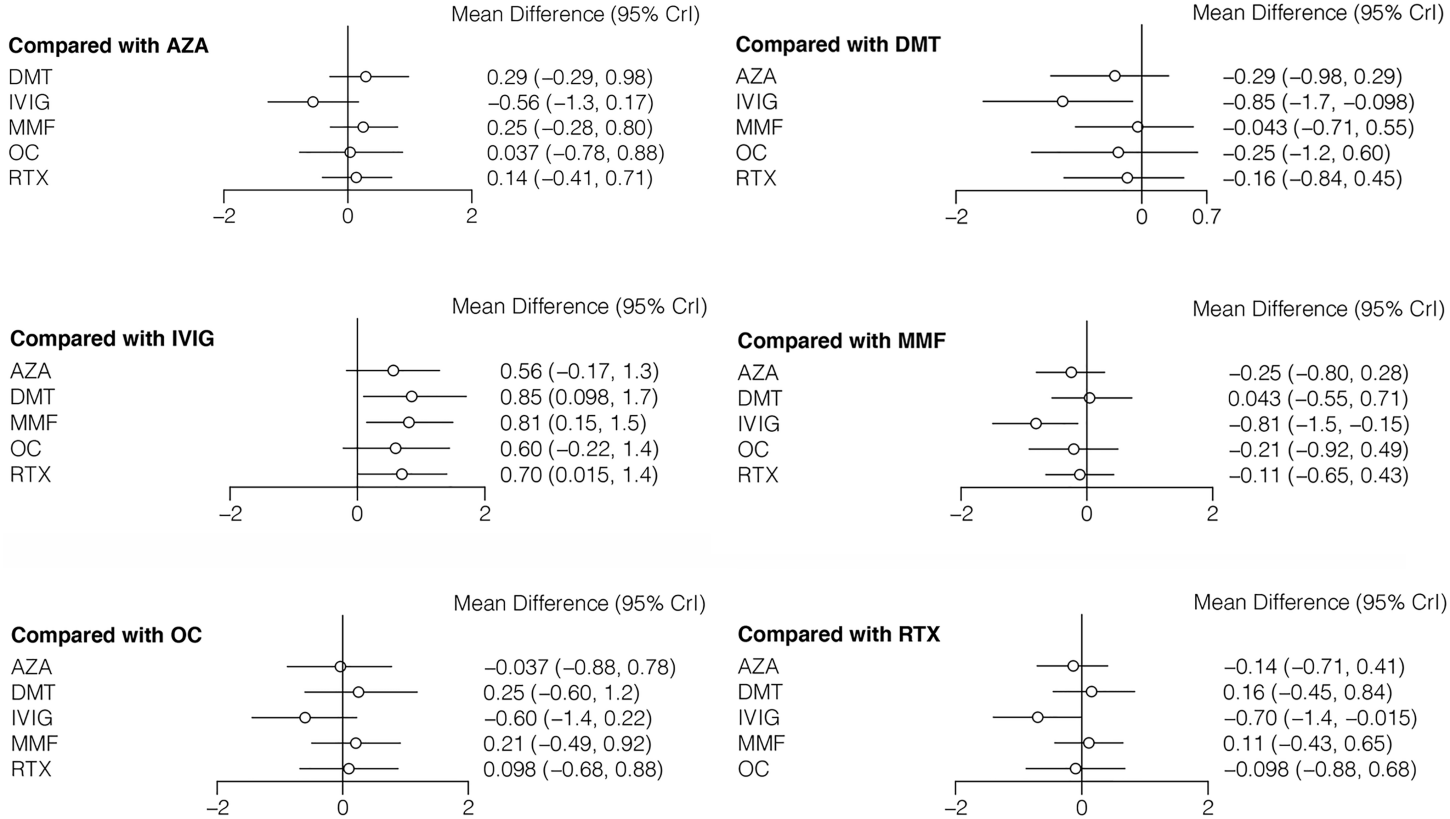
Forest plots of network meta-analysis for annualized relapse rate.

**Figure 4:**
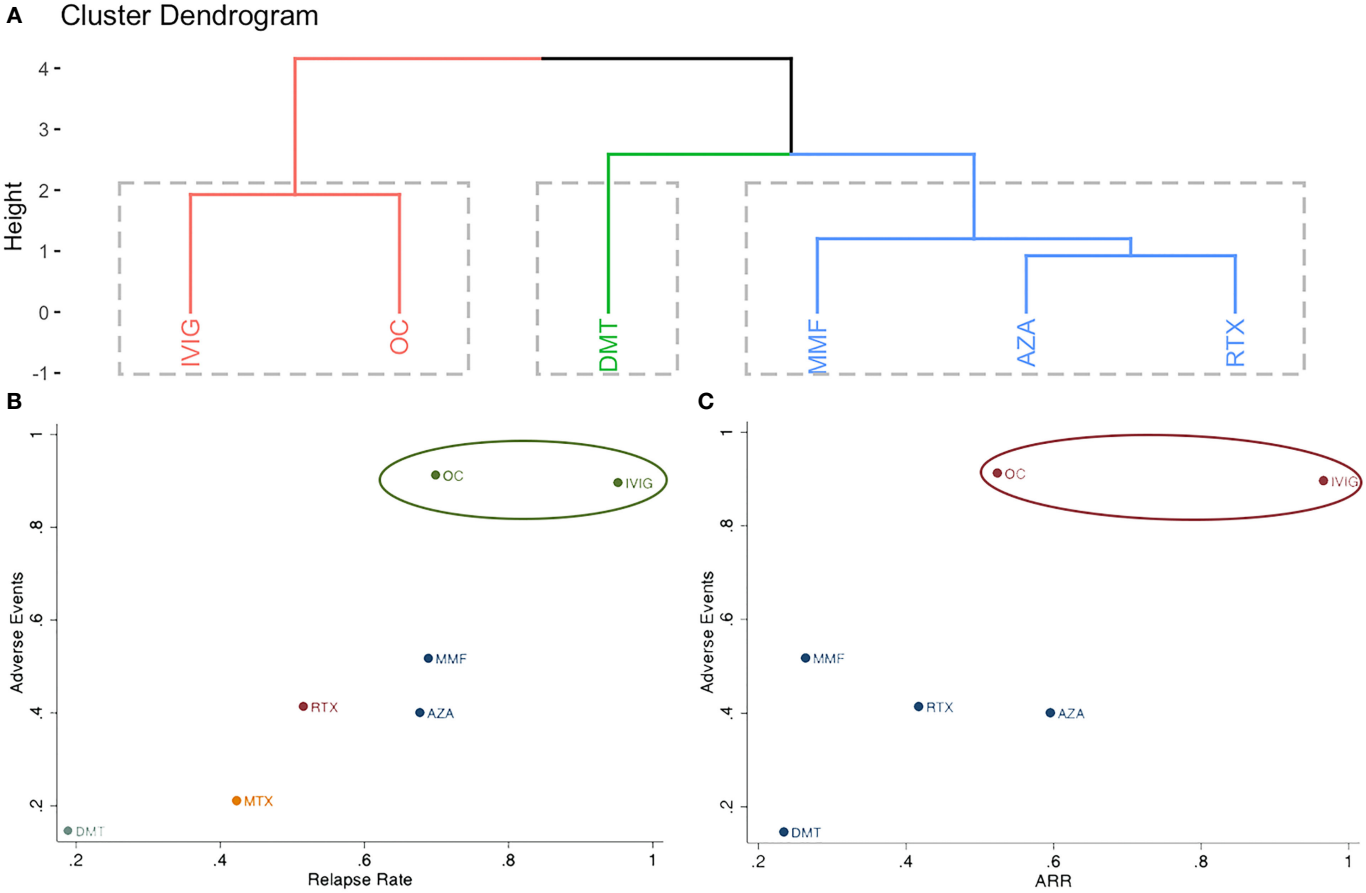
Clustering analysis of different treatments for **(A)**: incidence of relapse, annualized relapse rate and adverse events; **(B)**: incidence of relapse and adverse events; **(C)**: annualized relapse rate and adverse events.

The ARR (with median and range or mean and SD) was reported in seven studies including six different treatments (AZA, DMT, IVIG, MMF, OC, and RTX) (7, 9, 11, 13–16). The mean differences with 95% CrIs of relative effectiveness are listed in **Table 3**. According to the SUCRA, IVIG was hierarchically superior to other treatments, followed by AZA, OC, RTX, MMF, and DMT (**eTable 2** in the **Supplement**). A comparison between different medications is shown in **Figure 3**. Compared with DMT (the last rank in SUCRA), IVIG significantly reduced the ARR (MD=−0.85, 95% CrI: −to −0.098). Compared with IVIG (the first rank in SUCRA), all other treatments showed a larger ARR, but significant differences were only observed in DMT, MMF (MD=0.81, 95% CrI: 0.15 to 1.5), and RTX (MD=0.70, 95% CrI: 0.015 to 1.4) (**Figure 4**). The node-splitting model exhibited segmental inconsistency for IVIG versus DMT (direct: MD=−1.9, 95% CrI: −3.0 to −0.7; indirect: MD=−0.22, 95% CrI: −1.2 to 0.7; network: MD=−0.85, 95% CrI: −1.7 to −0.1; *P*=.04), but all results showed superiority of IVIG compared with DMT. Network meta-regression was performed to adjust the influence of age, sample size, and risk of bias, and most treatments were not significantly affected by these modifiers, except for IVIG, which showed better results in adult patients than in children (**eTable 4** in the **Supplement**).

**Table 3:** Estimated differences in the efficacy of treatments in reducing the annualized relapse rate in MOG-AD.

AZA	0.29 (-0.29, 0.98)	-0.56 (-1.29, 0.17)	0.25 (-0.28, 0.80)	0.037 (-0.78, 0.88)	0.14 (-0.41, 0.71)
-0.29 (-0.98, 0.29)	DMT	-0.85 (-1.71, -0.098)	-0.043 (-0.71, 0.55)	-0.25 (-1.19, 0.60)	-0.16 (-0.84, 0.45)
0.56 (-0.17, 1.29)	0.85 (0.098, 1.71)	IVIG	0.81 (0.15, 1.50)	0.60 (-0.22, 1.44)	0.70 (0.015, 1.40)
-0.25 (-0.80, 0.28)	0.043 (-0.55, 0.71)	-0.81 (-1.50, -0.15)	MMF	-0.21 (-0.92, 0.49)	-0.11 (-0.65, 0.43)
-0.037 (-0.88, 0.78)	0.25 (-0.60, 1.19)	-0.60 (-1.44, 0.22)	0.21 (-0.49, 0.92)	OC	0.098 (-0.68, 0.88)
-0.14 (-0.71, 0.41)	0.16 (-0.45, 0.84)	-0.70 (-1.40, -0.015)	0.11 (-0.43, 0.65)	-0.098 (-0.88, 0.68)	RTX

AZA, azathioprine; DMT, disease-modifying therapy; IVIG, intravenous immunoglobulins; MMF, mycophenolate mofetil; OC, oral corticosteroids; RTX, rituximab. A deeper color indicates statistical significance.

### Comparison of adverse events of different treatments

Adverse events were reported in three studies, including seven different treatments (AZA, OC, DMT, IVIG, MMF, MTX, and RTX) (6, 8, 14). The estimated ORs with 95% CrIs of relative effectiveness are listed in **eTable 5** in the **Supplement**. According to the SUCRA, OC was hierarchically superior to other treatments, followed by IVIG, MMF, RTX, AZA, MTX, and DMT (**eTable 2** in the **Supplement**). OC was the most effective treatment and significantly reduced the incidence of adverse events compared with other agents, except for IVIG (**eTable 5** in the **Supplement**). The combined results for relapse rate and adverse events, as well as ARR and adverse events are presented in **Figure 4** in the **Supplement**; IVIG and OC grouped in the same cluster and were the most effective and tolerable therapies.

### Quality assessment

It should be noted that our meta-analysis only included retrospective studies, so the highest confidence of evidence is “moderate,” followed by “low” and “very low.” According to the CINeMA, eight (25%) of 32 comparisons for the incidence of relapse were rated as moderate confidence of evidence, two (6.25%) as low, and 22 (68.75%) as very low (**eTable 6** in the **Supplement**). IVIG : NT and OC : NT were categorized as having moderate confidence in evidence.

## Discussion

This network meta-analysis was based on 12 retrospective studies that included 735 patients with MOG-AD assigned to 10 different therapies. To the best of our knowledge, this is the first study to compare the efficacy and tolerability of different therapies for MOG-AD in a Bayesian network meta-analysis. Our main findings revealed that IVIG may be the optimal treatment for MOG-AD, followed by OC. Additionally, AZA, MMF, and RTX were found to reduce the relapse rate compared with NT and may therefore be suitable alternative therapies. In contrast, as DMT may increase the relapse rate and ARR in patients with MOG-AD, its use should be limited.

The use of IVIG is more common in other CNS demyelinating diseases, such as MS, as compared to MOG-AD. Olyaeemanesh et al. (21) performed a meta-analysis including six randomized controlled trials to evaluate the efficacy of IVIG for the treatment of MS; they found that whilst IVIG significantly reduced the relapse rate, it might have no impact on Expanded Disability Status Scale scores or ARR. Lewańska et al. (22) and Kocer et al. (23) found that the monthly use of IVIG reduced the number of brain lesions. For MOG-AD, a recent multicenter study by Chen et al. (24) showed that maintenance IVIG was effective in reducing relapses. To date, IVIG has not been considered as a first- or second-line therapy for MOG-AD due to insufficient clinical evidence. The superiority of IVIG in treating MOG-AD, which diminished the relapse rate, has been demonstrated in this study. However, there is a lack of standard IVIG regimen. Ramanathan et al. (14) administered 2 g/kg IVIG during the induction phase followed by a monthly infusion of 1 g/kg, which is a higher dosage than that used for treating MS (usually 0.2 to 0.4 g/kg/month) but similar to that used for NMOSD (21, 25, 26). Chen et al. (24) reported 1 g/kg of IVIG every 4 weeks or more might be a proper dose. Future studies with standard regimens and strict research designs are needed to verify the advantages of IVIG in treating MOG-AD.

Maintenance OC have inhibitory effects on the human immune system, including the reduction of antibody titers and T cell numbers. The broad immunosuppressive effects of OC make it a widely used therapy for treating autoimmune diseases. Some studies have suggested that MOG-AD is a steroid-sensitive disease (9, 14). Therefore, OC may be a promising therapy for treating MOG-AD, which was corroborated in this study. On the other hand, patients might relapse upon rapid withdrawal or a reduction of the maintenance dose of OC (27), and long-term use of OC may have side effects including infection, cushingoid features, hypertension, diabetes, and osteopenia. Therefore, additional immunosuppressive therapies such as AZA and MMF are frequently needed.

Both AZA and MMF prohibit the cell cycle of T cells from the S to G_2_ stage; therefore, treatment with these drugs would be expected to yield results similar to those obtained with OC therapy. AZA and MMF are off-label second-line treatments for NMOSD. Our previous study regarding the efficacy of different immunotherapies for NMOSD showed that the two drugs significantly reduced the ARR and Expanded Disability Status Scale scores compared with those obtained in response to OC or NT (28). Here, we confirmed that the two immunosuppressors had similar efficacy in preventing relapse of MOG-AD compared with that of OC and showed obvious superiority over NT. However, it should be noted that the combined use of AZA and MMF with OC may affect the curative effect. Therefore, the results of this meta-analysis should be interpreted with caution.

RTX is one of the most commonly used therapies for treating MOG-AD. A recent network meta-analysis showed that RTX ranked first among five different immunosuppressants for NMOSD (29). However, the efficacy of RTX for MOG-AD is not as good as expected (30), which may be based on several possible reasons. First, even in NMOSD, some patients show resistance to RTX, which may be due to the persistence of long-lived CD19^+^ cell clusters. By using CD19 antibodies, such as inebilizumab, or blocking the interleukin-6 receptor signaling pathway, the number of CD19^+^ B cells can be reduced, and patients with NMOSD and MOG-AD may have fewer relapses. Second, the pathogenesis of MOG-AD and NMOSD is different (1): studies have found that both IgG titers and complement deposition are less pronounced in MOG-AD than in NMOSD. The internalization or downregulation of MOG may also be involved in the pathogenesis. The above evidence may account for the larger proportion of treatment failure in MOG-AD than in NMOSD. In summary, RTX is not an optimal therapy for MOG-AD.

Except for the abovementioned common treatments, TAC, MTX, and CTX were prescribed to several MOG-AD patients. TAC blocks the Ca^2+^-calcineurin-NFAT pathway and inhibits the activation of T cells and has been used for the treatment of autoimmune diseases such as rheumatoid arthritis, systemic lupus erythematosus, psoriasis, and myasthenia gravis. However, in this meta-analysis, TAC was found to be associated with an increased risk of relapse, and the CrIs in the effectiveness of TAC were rather large, which may be due to the small sample size. Anticarcinogens, such as MTX and CTX, can induce immunogenic cell death (31, 32), which can increase the function of cytotoxic T cells and activate the type I interferon signaling pathway; thus, this phenomenon may explain the treatment failure of these two drugs in MOG-AD. In a network meta-analysis performed by Huang et al, CTX was the worst therapy for NMOSD. Combining the above evidence, MTX and CTX should be used with caution.

DMTs (including interferon-β, glatiramer acetate, natalizumab, teriflunomide, fingolimod, and mitoxantrone) were also used in a small number of patients with MOG-AD. Our meta-analysis found that considering efficacy and tolerance, DMT had the lowest ranking amongst the analyzed treatments. This result illustrates the difference in the pathogenesis of MOG-AD and MS. Based on our results, we recommend avoiding the use of DMT in treating MOG-AD.

There are some limitations to this study. Traditionally, only randomized controlled trials or prospective comparative studies are included in network meta-analyses. However, these types of studies are rare in the field of MOG-AD. The methods of calculating ARR were not always the same in retrospective studies. The EDSS was not reported as a primary outcome in all studies. As the exclusive consideration of retrospective studies would have negatively affected the quality of this study, we performed a quality assessment and found that IVIG versus NT and OC versus NT had the highest confidence of evidence. In addition, we performed a meta-regression analysis to adjust for confounding factors such as age and sample size. The second limitation is that some studies with large sample sizes and a lack of comparative groups were not included, for example, Ringelstein et al. (33) found blocking IL-6R was safe and effective in treating MOG-AD; Chen et al. and Hacohen et al. (24) recently performed a multicenter cohort study and confirmed the efficacy of IVIG in treating MOG-AD; therefore, the exclusion of single-arm studies may exaggerate or underestimate the efficacy of certain treatments. Third, the definite therapeutic efficacy could not be assessed due to the combination of immunosuppressors with OC in some studies, and many of the studies have included patients more than once if they have been on multiple treatments. Forth, most of the studies included only patients who had a relapsing disease and were commenced on maintenance immunotherapy but not all. The ARR before treatment was lower in the NT group in some studies (8, 10). This may also underestimate the efficacy of certain treatments.

Despite the limitations, our meta-analysis of 735 patients provided the first comprehensive comparison between MOG-AD treatments. Altogether, our results suggest that IVIG and OC are superior to other immunosuppressants and could be used as first-line therapies. AZA, MMF, and RTX reduce the relapse rate compared with NT, rendering them viable alternative treatments. Importantly, DMT should not be used as a common treatment for MOG-AD.

## Conclusion

Treatment with IVIG, in addition to OC, significantly reduced the ARR and caused few adverse events. RTX, MMF, and AZA also showed good efficacy and tolerability. Whilst DMT should be avoided, IVIG and OC may be suited as first-line therapies for patients with MOG-AD. RTX, MMF, and AZA present suitable alternatives.

## Data Availability Statement

The original contributions presented in the study are included in the article/**Supplementary Material**. Further inquiries can be directed to the corresponding author.

## Author Contributions

XW: methodology, software, formal analysis, writing-original draft. LK: writing-reviewing and editing, methodology, validation. ZZ: formal analysis, writing-original draft. ZS and HC: formal analysis, methodology, validation. YL and QD: methodology. HZ: conceptualization, validation, investigation, writing-review and editing, supervision. All authors contributed to the article and approved the submitted version.

## Funding

This work was funded by the Natural Science Foundation of Sichuan Province (Grant No. 2022NSFSC1591 to ZZ and 2022NSFSC1432 to XW), Department of Science and Technology of Sichuan Province (Grant No. 2021YFS0173 to HZ), and 1·3·5 project for disciplines of excellence–Clinical Research Incubation Project, West China Hospital, Sichuan University (Grant No. 21HXFH041 to HZ).

## Acknowledgments

We would like to thank Editage (www.editage.cn) for English language editing.

## Conflict of Interest

The authors declare that the research was conducted in the absence of any commercial or financial relationships that could be construed as a potential conflict of interest.

## Publisher’s Note

All claims expressed in this article are solely those of the authors and do not necessarily represent those of their affiliated organizations, or those of the publisher, the editors and the reviewers. Any product that may be evaluated in this article, or claim that may be made by its manufacturer, is not guaranteed or endorsed by the publisher.
