# Supplementary material for: Effectiveness and tolerability of different therapies in preventive treatment of MOG-IgG-associated disorder: A network meta-analysis
Source: Front Immunol. 2022 Jul 26;13:953993. doi: 10.3389/fimmu.2022.953993 (PMC9360318; doi:10.3389/fimmu.2022.953993)
Supplement: Supplementary file 1 [file Table_1.docx]

**eTable 1. Search strategies for the network meta-analysis.**

|  | Terms | Number |
| --- | --- | --- |
| ***Pubmed*** | | |
| 1 | "myelin oligodendrocyte glycoprotein"[Title/Abstract] OR "oligodendrocyte myelin glycoprotein"[MeSH Terms] OR "myelin oligodendrocyte glycoprotein"[MeSH Terms] OR "mog"[Title/Abstract] OR "mogad"[Title/Abstract] | 4,557 |
| 2 | "treatment"[Title/Abstract] OR "outcome"[Title/Abstract] OR "effect"[Title/Abstract] OR "therapy"[Title/Abstract] OR "therapeutic"[Title/Abstract] OR "efficacy"[Title/Abstract] | 9,342,556 |
| 3 | #1 AND #2 | 2,186 |
| Embase | | |
| 1 | 'myelin oligodendrocyte glycoprotein'/exp | 4,661 |
| 2 | 'mogad' | 133 |
| 3 | 'mog-ad' | 16 |
| 4 | 'mog-igg' | 439 |
| 5 | #1 OR #2 OR #3 OR #4 | 4,843 |
| 6 | 'treatment'/exp | 5,831 |
| 7 | 'outcome' /exp | 40 |
| 8 | 'effect' | 6,032,525 |
| 9 | 'therapy' | 8,862,531 |
| 10 | 'therapeutic' | 1,679,407 |
| 11 | 'efficacy'/exp | 927 |
| 12 | #6 OR #7 OR #8 OR #9 OR #10 OR #11 | 13,261,019 |
| 13 | #5 AND #12 | 2,467 |
| Web of science | | |
| 1 | TS=myelin oligodendrocyte glycoprotein | 6,542 |
| 2 | TS=mogad | 109 |
| 3 | TS=mog-ad | 16 |
| 4 | TS=mog-igg | 350 |
| 5 | #1 OR #2 OR #3 OR #4 | 6,629 |
| 6 | TS=treatment | 11,525,291 |
| 7 | TS=outcome | 3,607,617 |
| 8 | TS=effect | 24,545,594 |
| 9 | TS=therapy | 10,404,296 |
| 10 | TS=therapeutic | 6,097,478 |
| 11 | TS=efficacy | 1,832,734 |
| 12 | #6 OR #7 OR #8 OR #9 OR #10 OR #11 | 37,240,218 |
| 13 | #5 AND #12 | 4,007 |
| Cochrane library | | |
| 1 | myelin oligodendrocyte glycoprotein | 31 |
| 2 | mogad | 1 |
| 3 | mog-ad | 0 |
| 4 | mog-igg | 0 |
| 5 | #1 OR #2 OR #3 OR #4 | 31 |
| 6 | treatment | 794,343 |
| 7 | outcome | 525,278 |
| 8 | effect | 533,324 |
| 9 | therapy | 720,102 |
| 10 | therapeutic | 298,260 |
| 11 | efficacyc | 376,395 |
| 12 | #6 OR #7 OR #8 OR #9 OR #10 OR #11 | 1,360,835 |
| 13 | #5 AND #12 | 28 |
