# Supplementary material for: Effectiveness and tolerability of different therapies in preventive treatment of MOG-IgG-associated disorder: A network meta-analysis
Source: Front Immunol. 2022 Jul 26;13:953993. doi: 10.3389/fimmu.2022.953993 (PMC9360318; doi:10.3389/fimmu.2022.953993)
Supplement: Supplementary file 2 [file Table_2.docx]

**eTable 2. The surface under the cumulative ranking curve (SUCRA) of different treatment methods.**

| Treatment | Incidence of relapse | Annualized relapse rate | Adverse event |
| --- | --- | --- | --- |
| AZA | 0.67682722 | 0.5955005 | 0.4009571 |
| CTX | 0.53292361 | - | - |
| DMT | 0.18850972 | 0.2343575 | 0.1463908 |
| IVIG | 0.95172194 | 0.9656165 | 0.8965821 |
| MMF | 0.68858333 | 0.2638115 | 0.5179288 |
| MTX | 0.42262583 | - | 0.2110492 |
| NT | 0.24273222 | - | - |
| OC | 0.69865778 | 0.5237315 | 0.9128808 |
| RTX | 0.51508889 | 0.4169825 | 0.4142113 |
| TAC | 0.08232944 | - | - |

AZA: azathioprine, CTX: cyclophosphamide, DMT: disease-modifying therapy, IVIG: intravenous immunoglobulins, MMF: mycophenolate mofetil, MTX: methotrexate, NT: no treatment, OC: oral corticosteroids, RTX: rituximab, TAC: tacrolimus
