# Supplementary material for: Effectiveness and tolerability of different therapies in preventive treatment of MOG-IgG-associated disorder: A network meta-analysis
Source: Front Immunol. 2022 Jul 26;13:953993. doi: 10.3389/fimmu.2022.953993 (PMC9360318; doi:10.3389/fimmu.2022.953993)
Supplement: Supplementary file 3 [file Table_3.docx]

**eTable 3. Network meta-regression of each treatment in reducing relapse rate compared with no treatment (NT) group.**

| Characteristics | All studies | Age | Sample size | Risk of bias |
| --- | --- | --- | --- | --- |
| AZA | 0.23  (0.063, 0.67) | 0.23  （0.066, 0.63) | 0.24  (0.063, 0.73) | 0.04  (3.21×10^-14^, 0.30) |
| CTX | 0.35  (0.014, 15.70) | 0.34  (0.016, 14.25) | 0.35  (0.0134, 16.45) | 0.045  (4.42×10^-14^, 3.60) |
| DMT | 1.33  (0.31, 5.0) | 1.39  (0.35, 4.88) | 1.42  (0.32, 5.65) | 0.23  (1.88×10^-13^, 2.01) |
| IVIG | 0.075  (0.016, 0.31) | 0.078  (0.017, 0.30) | 0.078  (0.016, 0.34) | 0.013  (1.14×10^-14^, 0.13) |
| MMF | 0.22  (0.062, 0.64) | 0.21  (0.062, 0.57) | 0.22  (0.059, 0.68) | 0.039  (2.98×10^-14^, 0.28) |
| MTX | 0.57  (0.031, 25.58) | 0.55  (0.033, 22.68) | 0.60  (0.030, 27.16) | 0.07  (7.08×10^-14^, 5.1) |
| OC | 0.21  (0.05, 0.80) | 0.20  (0.05, 0.71) | 0.21  (0.048, 0.85) | 0.035  (2.85×10^-14^, 0.31) |
| RTX | 0.34  (0.10, 0.98) | 0.31  (0.10, 0.85) | **0.34**  **(0.10, 1.06)** | 0.051  (4.11×10^-14^, 0.37) |
| TAC | 5.85  (0.19, 316.83) | 5.9  (0.21, 299.77) | 6.04  (0.19, 339.19) | 0.77  (8.04×10^-13^, 74.06) |

AZA: azathioprine, CTX: cyclophosphamide, DMT: disease-modifying therapy, IVIG: intravenous immunoglobulins, MMF: mycophenolate mofetil, MTX: methotrexate, NT: no treatment, OC: oral corticosteroids, RTX: rituximab, TAC: tacrolimus
