# Supplementary material for: Effectiveness and tolerability of different therapies in preventive treatment of MOG-IgG-associated disorder: A network meta-analysis
Source: Front Immunol. 2022 Jul 26;13:953993. doi: 10.3389/fimmu.2022.953993 (PMC9360318; doi:10.3389/fimmu.2022.953993)
Supplement: Supplementary file 4 [file Table_4.docx]

**eTable 4. Network meta-regression of each treatment in reducing annualized relapse rate compared with disease modified therapy (DMT) group.**

| Characteristics | All studies | Age | Sample size | Risk of bias |
| --- | --- | --- | --- | --- |
| AZA | -0.29  (-0.98, 0.29) | -0.29  （-1.10, 0.42) | -0.39  (-1.10, 0.21) | -0.39  (-1.10, 0.21) |
| IVIG | -0.85  (-1.70, -0.098) | **-0.85**  **(-1.80, 0.034)** | -0.91  (-1.70, -0.16) | -0.91  (-1.70, -0.16) |
| MMF | -0.043  (-0.71, 0.60) | -0.04  (-0.80, 0.68) | -0.12  (-0.77, 0.49) | -0.12  (-0.77, 0.49) |
| OC | -0.25  (-1.20, 0.60) | -0.25  (-1.30, 0.72) | -0.32  (-1.20, 0.53) | -0.32  (-1.20, 0.53) |
| RTX | -0.16  (-0.84, 0.45) | -0.15  (-0.92, 0.57) | -0.23  (-0.89, 0.39) | -0.23  (-0.89, 0.39) |

AZA: azathioprine, DMT: disease-modifying therapy, IVIG: intravenous immunoglobulins, MMF: mycophenolate mofetil, OC: oral corticosteroids, RTX: rituximab
