# Supplementary material for: Effectiveness and tolerability of different therapies in preventive treatment of MOG-IgG-associated disorder: A network meta-analysis
Source: Front Immunol. 2022 Jul 26;13:953993. doi: 10.3389/fimmu.2022.953993 (PMC9360318; doi:10.3389/fimmu.2022.953993)
Supplement: Supplementary file 5 [file Table_5.docx]

**eTable 5. Comparision of adverse events of different treatments in MOG-AD.**

| **AZA** | 14.28  (0.048, 12995.40) | 1.21e-15  (7.45e-51, 0.70) | 0.33  (0.0025, 48.75) | 7.99  (0.015, 9044.99) | 3.09e-16  (8.22e-51, 0.093) | 0.90  (0.015, 90.23) |
| --- | --- | --- | --- | --- | --- | --- |
| 0.070  (7.70e-05, 20.82) | **DMT** | 6.76e-17  (3.76e-52, 0.089) | 0.022  (1.34e-05, 18.90) | 0.55  (0.0010, 214.95) | 1.80e-17  (3.52e-52, 0.011) | 0.061  (0.00014, 17.40) |
| 8.28e+14  (1.43, 1.34e+50) | 1.48e+16  (11.23, 2.66e+51) | **IVIG** | 2.83e+14  (0.39, 4.96e+49) | 7.67e+15  (5.26, 1.52e+51) | 0.21  (2.49e-39, 1.21e+38) | 8.06e+14  (1.24, 1.43e+50) |
| 2.99  (0.021, 403.99) | 44.99  (0.053, 74577.86) | 3.53e-15  (2.02e-50, 2.55) | **MMF** | 24.61  (0.018, 51326.37) | 9.13e-16  (2.21e-50, 0.30) | 2.72  (0.028, 367.23) |
| 0.13  (0.00011, 66.77) | 1.82  (0.0047, 976.49) | 1.30e-16  (6.59e-52, 0.19) | 0.041  (1.95e-05, 56.69) | **MTX** | 3.29e-17  (7.05e-52, 0.023) | 0.11  (0.00020, 56.65) |
| 3.24e+15  (10.73, 1.22e+50) | 5.56e+16  (92.41, 2.84e+51) | 4.76  (8.24e-39, 4.01e+38) | 1.09e+15  (3.30, 4.53e+49) | 3.04e+16  (42.73, 1.42e+51) | **OC** | 3.20e+15  (10.07, 1.28e+50) |
| 1.11  (0.011, 66.53) | 16.30  (0.057, 7066.92) | 1.24e-15  (7.01e-51, 0.81) | 0.37  (0.0027, 35.16) | 8.96  (0.018, 5075.52) | 3.13e-16  (7.83e-51, 0.099) | **RTX** |

AZA: azathioprine, DMT: disease-modifying therapy, IVIG: intravenous immunoglobulins, MMF: mycophenolate mofetil, MTX: methotrexate, OC: oral corticosteroids, RTX: rituximab. A deeper color indicates statistical significance.
